# Temperature‐Dependent Electronic Ground‐State Charge Transfer in van der Waals Heterostructures

**DOI:** 10.1002/adma.202008677

**Published:** 2021-05-25

**Authors:** Soohyung Park, Haiyuan Wang, Thorsten Schultz, Dongguen Shin, Ruslan Ovsyannikov, Marios Zacharias, Dmitrii Maksimov, Matthias Meissner, Yuri Hasegawa, Takuma Yamaguchi, Satoshi Kera, Areej Aljarb, Mariam Hakami, Lain‐Jong Li, Vincent Tung, Patrick Amsalem, Mariana Rossi, Norbert Koch

**Affiliations:** ^1^ Advanced Analysis Center Korea Institute of Science and Technology (KIST) Seoul 02792 South Korea; ^2^ Fritz Haber Institute of the Max Planck Society 14195 Berlin Germany; ^3^ Chaire de Simulation à l'Echelle Atomique (CSEA) Ecole Polytechnique Fédérale de Lausanne (EPFL) Lausanne CH‐1015 Switzerland; ^4^ Humboldt‐Universität zu Berlin Institut für Physik and IRIS Adlershof 12489 Berlin Germany; ^5^ Helmholtz‐Zentrum für Materialien und Energie GmbH 12489 Berlin Germany; ^6^ Department of Mechanical and Materials Science Engineering Cyprus University of Technology Limassol 3603 Cyprus; ^7^ Max Planck Institute for the Structure and Dynamics of Matter 22761 Hamburg Germany; ^8^ Institute for Molecular Science Okazaki 444‐8585 Japan; ^9^ Physical Sciences and Engineering King Abdullah University of Science and Technology Thuwal 23955‐6900 Saudi Arabia; ^10^ Department of Mechanical Engineering The University of Hong Kong Pok Fu Lam Road Hong Kong China

**Keywords:** 2D semiconductors, charge transfer, electron–phonon coupling, molecular dopants, MoS _2_, photoelectron spectroscopy

## Abstract

Electronic charge rearrangement between components of a heterostructure is the fundamental principle to reach the electronic ground state. It is acknowledged that the density of state distribution of the components governs the amount of charge transfer, but a notable dependence on temperature is not yet considered, particularly for weakly interacting systems. Here, it is experimentally observed that the amount of ground‐state charge transfer in a van der Waals heterostructure formed by monolayer MoS_2_ sandwiched between graphite and a molecular electron acceptor layer increases by a factor of 3 when going from 7 K to room temperature. State‐of‐the‐art electronic structure calculations of the full heterostructure that accounts for nuclear thermal fluctuations reveal intracomponent electron–phonon coupling and intercomponent electronic coupling as the key factors determining the amount of charge transfer. This conclusion is rationalized by a model applicable to multicomponent van der Waals heterostructures.

## Graphical abstract

The amount of charge transfer between components of a van der Waals heterostructure to reach the electronic ground state can exhibit a strong temperature dependence. For a donor–bridge–acceptor‐type heterostructure, the intra component electron–phonon coupling and the intercomponent electronic coupling result in a temperature‐dependent density of states available for charge transfer.

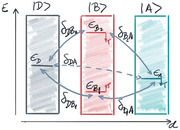

## 1 Introduction

The discovery that atomically thin layers can exist at room temperature in air marked the launch of research on 2D materials.^[^ ^1^, ^2^ ^]^ A single layer of a 2D material may be insulating (e.g., hexagonal boron nitride), semiconducting (e.g., MoS_2_), or conducting (e.g., graphene), and thus all electrical material properties required for the construction of an electronic or optoelectronic device are available in the monolayer limit.^[^ ^3^, ^4^, ^5^, ^6^ ^]^ Stacks of such monolayers are typically bound by weak van der Waals (vdW) interlayer interactions, and vdW heterostructures have emerged as prime candidates for realizing electronic and optoelectronic functions in the smallest possible volume. The type and efficiency of the functionality that can be achieved depends critically on the electronic energy level alignment across the heterostructure,^[^ ^7^ ^]^ and substantial charge density rearrangement upon contact can occur, depending on the electronic structure of each component. Consequently, charge rearrangement and also charge transfer (CT) phenomena that define the electronic ground state of vdW heterostructures must be thoroughly understood for knowledge‐guided device design. Beyond layered inorganic 2D materials, organic molecular semiconductors are also attractive as a component in vdW heterostructures, because they extend the range of available energy gap values and feature strong light–matter interaction.^[^ ^8^, ^9^ ^]^ Furthermore, it has been recognized that strong molecular electron acceptors and donors can be employed as dopants for numerous 2D semiconductors, particularly for transition metal dichalcogenides (TMDCs).^[^ ^10^, ^11^, ^12^, ^13^, ^14^ ^]^ This doping, on the one hand, allows controlling the Fermi level (*E*_F_) position and mobile carrier density in the semiconductor, and, on the other hand, enables manipulation of optical quasiparticles, such as charged excitons (positive or negative trions) in TMDC monolayers.^[^ ^15^, ^16^, ^17^, ^18^, ^19^ ^]^ Such vdW heterostructures can be ideal model systems for exploring intriguing physical phenomena,^[^ ^20^, ^21^ ^]^ and they can pave the way for a broader class of device applications.

**Figure 1:**
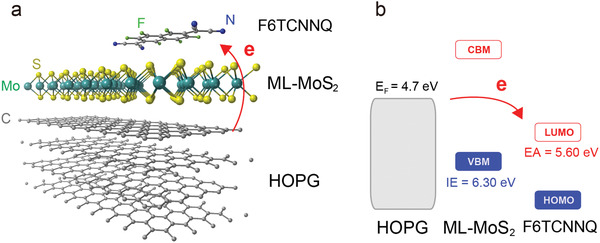
Ground‐state charge transfer in the vdW heterostructure F6TCNNQ/ML‐MoS_2_/HOPG. a) Schematic configuration and b) energy level diagram of the investigated F6TCNNQ/ML‐MoS_2_/HOPG van der Waals heterostructure.

Despite many opportunities offered by vdW heterostructures, the current understanding of contact‐induced charge density rearrangement phenomena and interlayer ground‐state CT is not well established for such weakly bound stacks, mostly due to their challenging complexity. For instance, organic semiconductors feature vast spatial degrees of freedom, and 2D semiconductors experience substantial band structure renormalization as function of the thermodynamic conditions^[^ ^22^, ^23^ ^]^ and of the surrounding environment, including a supporting substrate.^[^ ^11^ ^]^ A recent study on vdW heterostructures comprising the molecular electron acceptor 1,3,4,5,7,8‐hexafluoro‐tetra‐cyano‐naphthoquinodimethane (F6TCNNQ) deposited on monolayer (ML) MoS_2_ revealed the key role of the substrate.^[^ ^11^ ^]^ When insulating sapphire was used as substrate for ML‐MoS_2_, electrons were transferred from n‐type gap states (induced by native sulfur vacancies) to molecular acceptors. In contrast, when sapphire was replaced by highly oriented pyrolytic graphite (HOPG), the n‐type gap states of MoS_2_ were emptied into the charge reservoir of the conductive substrate and integer one‐electron transfer took place directly from HOPG to F6TCNNQ, as schematically shown in **Figure** **1**. This scenario was discussed in analogy to a parallel plate capacitor, where positive and negative charges reside on the two “plates,” HOPG and F6TCNNQ, respectively. The ML‐MoS_2_ then represents a dielectric layer experiencing the electric field between the plates, and in this model the frontier energy levels of the semiconductor are not involved in CT. Considering only this picture, the amount of charge transferred across the vdW heterostructure, i.e., the number of molecules that receive an electron and become an anion, to reach electronic equilibrium is determined by the density of state (DOS) distribution near *E*_F_ in HOPG and that of the lowest unoccupied molecular orbital (LUMO) level manifold in F6TCNNQ, as well as their energy offset. However, conjugated molecules exhibit strong electron‐vibrational coupling, i.e., the electronic energy levels change upon intramolecular bond length alterations. This translates into a variation of the ground‐state electronic levels’ energy when vibrational modes are populated.^[^ ^24^, ^25^, ^26^ ^]^ Therefore, the temperature‐dependent population of vibrational modes is expected to alter significantly the electron distribution near *E*_F_ and hence the magnitude of CT in vdW heterostructures. This dependence of CT on temperature has not been investigated in vdW heterostructures to date. An indication of the relevance of temperature on CT is the observation that the electrical characteristics of ML‐MoS_2_‐based field‐effect transistors depend notably on temperature when a molecular acceptor is deposited onto the semiconductor.^[^ ^14^ ^]^ For molecular acceptors from the tetracarboxylic‐dianhydride family deposited on silver single crystals it was found that decreasing the temperature significantly increased the amount of CT, due to the shortening of the molecule–metal distance at lower temperature.^[^ ^27^, ^28^ ^]^ However, these systems are characterized by covalent interactions and pronounced orbital hybridization, which makes a direct relation to vdW heterostructures uncertain. In fact, for the vdW heterostructure F6TCNNQ/HOPG we find that the amount of CT is virtually independent of temperature (see Figure S1 in the Supporting Information).

Here, we demonstrate with angle‐resolved photoelectron spectroscopy (ARPES) that the amount of ground‐state CT in the prototypical vdW heterostructure F6TCNNQ/ML‐MoS_2_/HOPG increases with increasing temperature, gradually and reversibly. This unexpected phenomenon is rationalized via high level electronic structure calculations of the full heterostructure, which accounts for electron–phonon coupling in the adiabatic limit through sampling of stochastic phonon displacements at different temperatures. The calculations show a shift to lower energies and broadening of the acceptor's LUMO manifold with increasing temperature that enhances CT. Within a simple model, it is shown that even in the absence of large‐scale structural changes, the energy‐gap renormalization of MoS_2_ and F6TCNNQ with temperature can contribute to the CT enhancement. Therefore, the earlier suggested capacitor model falls short of correctly describing such systems, because it only considers electrostatic interaction.

## 2 Results

### 2.1 Temperature‐Dependent Charge Transfer in the F6TCNNQ/ML‐MoS_2_/HOPG vdW Heterostructure

**Figure 2:**
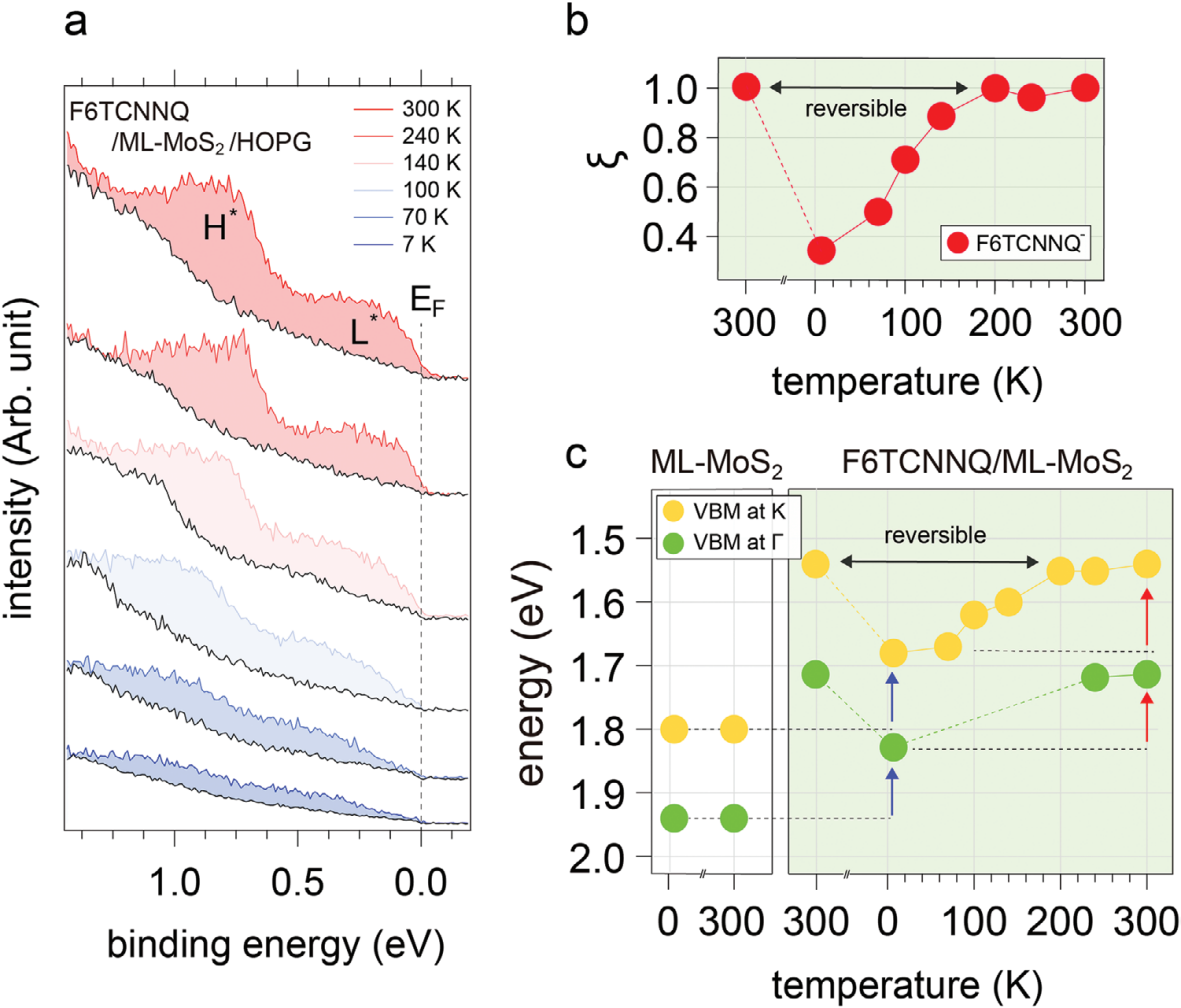
Temperature‐dependent ground‐state charge transfer in the vdW heterostructure. a) Energy distribution curves (EDCs) of MoS_2_/HOPG near the Fermi level as a function of temperature, before (black) and after (colored) 0.5 ML F6TCNNQ deposition. The additional density of states after F6TCNNQ deposition is due to negatively charged acceptors (F6TCNNQ^–^) only. b) Relative change in charged F6TCNNQ (ξ) as a function of temperature, where the temperature axis also reflects the experimental sequence, i.e., starting at 300 K, cooling to 7 K, followed by incremental return to 300 K, to highlight the reversibility. c) Valence band maximum (VBM) of MoS_2_ at the Γ and K points plotted as a function of temperature with/without absorption of F6TCNNQ. L^*^ and H^*^ denote the partially filled LUMO and relaxed HOMO of charged F6TCNNQ.^[^ ^11^ ^]^

ARPES allows tracking the valence band structure of ML‐MoS_2_ and the characteristic valence fingerprints of negatively charged F6TCNNQ molecules (anions).^[^ ^29^ ^]^ For a vdW heterostructure consisting of ≈0.5 ML F6TCNNQ on ML‐MoS_2_/HOPG (representative optical and atomic force microscopy images for ML‐MoS_2_/HOPG shown in Figures S2 and S3 in the Supporting Information) the corresponding energy distribution curves (EDCs) in the near‐*E*_F_ region from ARPES measurements are shown in **Figure** **2**a, for different temperatures. At room temperature (300 K), this energy region is dominated by two features centered at 0.2 and 0.8 eV binding energy (see Figure S5 in the Supporting Information), as the valence band onset of MoS_2_ is at a binding energy higher than 1.5 eV (see Figure S6 in the Supporting Information). These can readily be assigned to emission from the singly occupied former LUMO level of the F6TCNNQ anion (denoted as L*) and its relaxed highest occupied molecular orbital (HOMO) level (denoted as H*), due to an integer electron transfer from HOPG.^[^ ^11^ ^]^ Spectra recorded at lower temperature exhibit the same features, but with incrementally reduced intensity. We note that the same molecular anion emission features can be identified for F6TCNNQ deposited directly onto HOPG, as shown in Figure S1 in the Supporting Information, but no intensity variation as function of temperature was observed. The acceptor molecules adopt a face‐on lying orientation on MoS_2_ supported by HOPG from room temperature to 77 K, as determined from X‐ray absorption measurements (see Figure S7 in the Supporting Information), and it is reasonable to assume that this configuration persists even at lower temperatures. Consequently, the area of the emission features L* and H* is representative of the fraction of molecular anions on the surface. Plotting this area ξ, normalized to the signal at 300 K, as function of temperature (Figure 2b), reveals a gradual decrease in the range between room temperature and 7 K. This is a direct indication that the fraction of charged F6TCNNQ molecules at 7 K is only about one third of that found at 300 K. The fraction of charged molecules at 300 K was calculated from the transferred electron density divided by the surface molecule density of F6TCNNQ and found to be 21.7%, while that at 7 K was 7.2% (for details see Section S3 and Table S1 in the Supporting Information for absolute charge density values). Since the shape of photoemission features from F6TCNNQ anions does not change, only its intensity, we conclude that the amount of CT per molecule—one electron—is independent of temperature. Note that the temperature‐dependent change of ξ is fully reversible, as the temperature sequence of this experiment started at 300 K, followed by cooling to 7 K, and subsequent warming‐up steps until reaching 300 K again. To provide further support for this finding of smaller CT amount at lower temperature, we analyze the corresponding MoS_2_ valence band maximum (VBM) positions at the K and Γ points of the ML's Brillouin zone in Figure 2c (selected band structure data plots are shown in Figure S6 in the Supporting Information). While the VBM positions of bare MoS_2_/HOPG show very little change (less than 50 meV) between 300 K and 7 K, they shift by up to 260 meV with F6TCNNQ on top. Furthermore, the trend of VBM shifts as function of temperature follows that already seen for ξ in Figure 2b.

These observations are consistent with the overall CT mechanism shown in Figure 1, i.e., electron transfer from HOPG to F6TCNNQ, whereas ML‐MoS_2_ experiences the electric field created by the separated charges and its energy level shift accordingly. The electric field is directly proportional to the amount of CT in the capacitor model, and the same temperature‐dependent trend for ξ and the VBM shifts is expected, and verified here by comparison of Figure 2b,c. For the vdW heterostructure investigated here, the position of *E*_F_ in the semiconductor gap can be tuned by ≈260 meV simply by varying the temperature, as the different CT amount changes the electric field from approximately 0.26 V nm^−1^ at 7 K to 0.57 V nm^−1^ at 300 K (estimated with an effective distance between HOPG and the molecular layer of 9 Å). The origin of the temperature‐dependent CT amount, however, remains to be elucidated. If lower temperature was to simply decrease nuclear motion and thus reduce the average vertical interatomic distances in the heterostructure, the CT amount would be expected to increase with decreasing temperature in the capacitor model; the opposite is observed in experiment. Therefore, to shed light on this observation, elaborate first‐principles modeling is employed to obtain more insight into how temperature influences the density of state (DOS) of F6TCNNQ/ML‐MoS_2_/HOPG.

### 2.2 Charge Transfer in vdW Heterostructures

**Figure 3:**
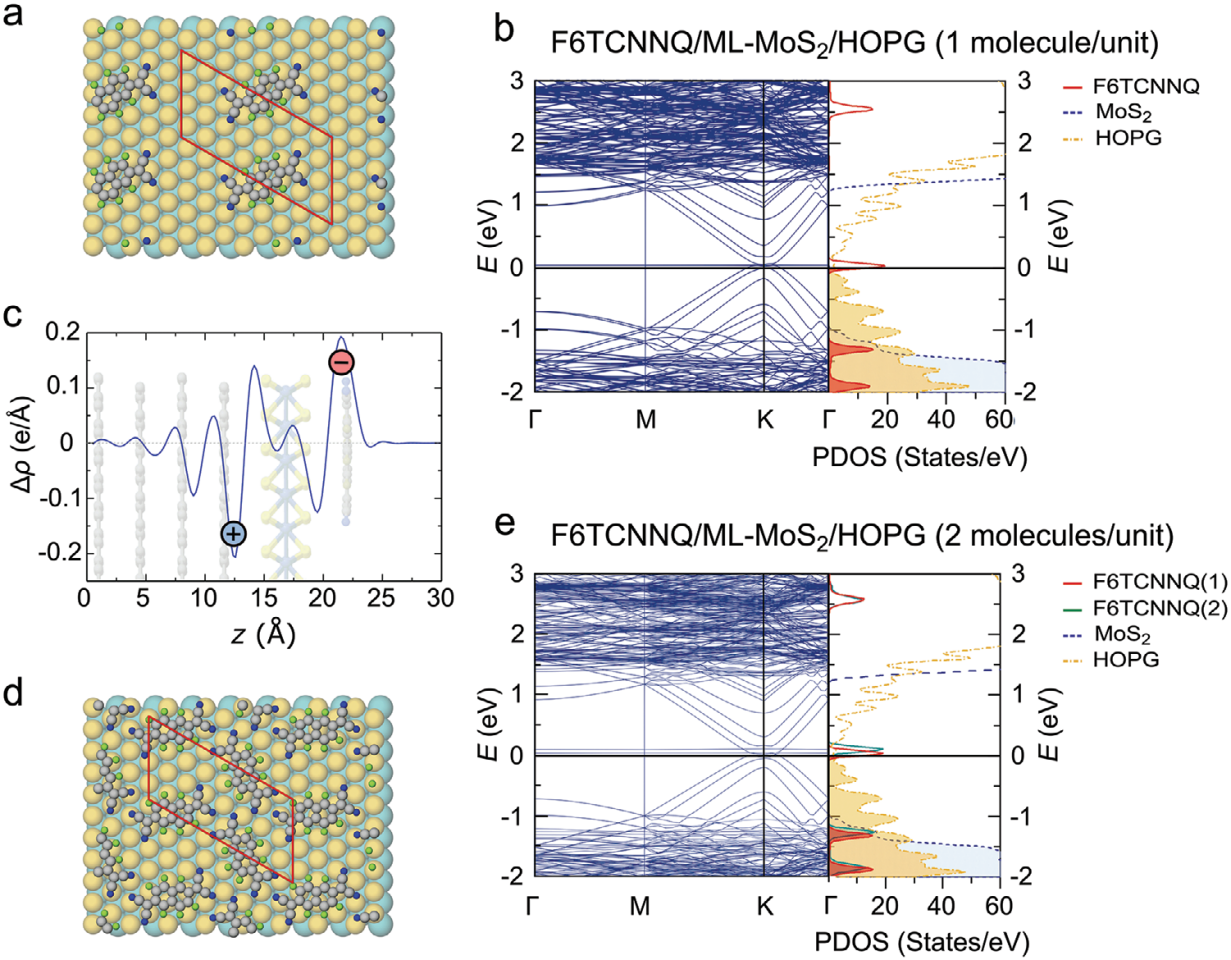
Calculated band structure of static F6TCNNQ/ML‐MoS_2_/HOPG heterostructures. a) Top view of dilute F6TCNNQ/ML‐MoS_2_(4 × 8)/HOPG. b) Band structure and PDOS (DFT‐HSE06) corresponding to the supercell shown in (a). The band structure is folded and PDOS and band structure have been shifted by 5.4 eV, such that the electronic occupation distribution drops below 0.09 above 0 eV (see the Experimental Section for details). c) Calculated plane average differential charge density ($\Delta \rho \;=\;\rho_{F6{\text{TCNNQ/MoS}}_{2}\text{/HOPG}}\;-\;\rho_{F6\text{TCNNQ}}-\rho_{{\text{MoS}}_{2}\text{/HOPG}}$) for the hybrid optimized structure. Positive values mean electron density accumulation (negative charge) while negative values mean electron density depletion (positive charge). d) Top view of 2 F6TCNNQ/ML‐MoS_2_(4 × 8)/HOPG. e) Band structure and PDOS (DFT‐HSE06) corresponding to the supercell shown in (d). The band structure is folded and PDOS and band structure have been shifted by 5.34 eV, such that the electronic occupation distribution drops below 0.09 above 0 eV.

We searched for stable structures of the vdW heterostructure composed of either one (dilute case) or two F6TCNNQ molecules on a 4 ×  8 ML‐MoS_2_ unit cell, which corresponds to almost the same molecular density as in experiments (see Section S3 in the Supporting Information). We employed density‐functional theory with a range‐separated hybrid functional (see computational details in the Experimental Section). Globally, the acceptor molecules adopt a flat‐lying orientation on ML‐MoS_2_ with a small corrugation of adsorption energy (maximum 150 meV) with respect to lateral displacements (see Figure S8 and Table S2 in the Supporting Information). The most stable structures are displayed in **Figure** **3**a,d. This lying orientation of the molecules agrees with experiment (see Figure S7 in the Supporting Information). Molecules placed on free‐standing ML‐MoS_2_ are charge–neutral, as expected from the respective energy levels (cf. experimental values in Figure 1b and calculated projected DOS (PDOS) in Figure S9 in the Supporting Information). Upon adding a commensurate HOPG substrate (modeled by a 5 × 10 supercell with four layers, totaling 522 atoms in the model) to F6TCNNQ/ML‐MoS_2_, the molecules become negatively charged in this model. This observation is supported by the charge density rearrangement plotted in Figure 3c, and is consistent with the band structures and PDOS shown in Figure 3b, where the zero of energy crosses the PDOS of the molecular LUMO levels. In agreement with the capacitor model discussed above in relation to Figure 1b, the calculated plane‐averaged differential charge density (Δρ) plot (Figure 3c) reveals an accumulation of positive charge in HOPG and corresponding negative charge in the molecular layer, giving rise to the electric field drop across ML‐MoS_2_ that shifts its energy levels. The partially occupied LUMO level appears as an almost completely flat band, in Figure 3b,e. We confirm that this is the molecular LUMO by visualizing the orbital in space and comparing it to the orbital of the isolated molecule (see Figure S10 in the Supporting Information). We note that the orbital shows nonvanishing hybridization with ML‐MoS_2_. This is a direct manifestation of the finite electronic coupling between the two components, even if their interaction can be classified as van der Waals type.

In the dilute case, the LUMO is partially occupied by 0.3 *e*, whereas the partially occupied LUMO orbitals of the two nonequivalent molecules in the densely packed case are not equally charged, i.e., we find that one molecule presents a partial occupation of 0.25 *e* on its partially filled LUMO and the other 0.1 *e*. The fractional charge transfer observed here (including the disproportional CT for two molecules in the unit cell) obtained with a range‐separated hybrid functional, in contrast to the experimental evidence of integer charge transfer, is likely a consequence of the remaining electron delocalization error present in this functional, allied to the need of much larger supercells that are not computationally feasible to simulate integer CT.^[^ ^30^, ^31^ ^]^ With this static description (i.e., without temperature‐induced effects from nuclear motion) of the vdW heterostructure we obtain adequate qualitative agreement between experiment and theory. In the following, we continue by including quantum zero‐point motion and temperature‐dependent effects in our calculations.

### 2.3 Effect of Temperature on Charge Transfer in the vdW Heterostructure

**Figure 4:**
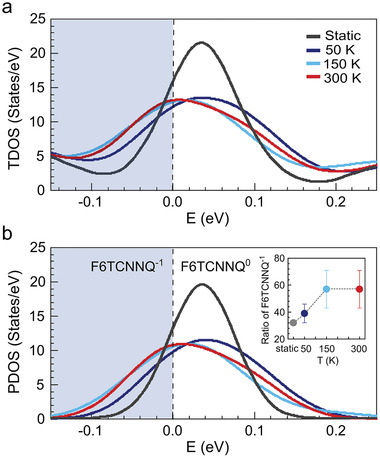
Nuclear thermal fluctuations and DOS near Fermi level. a) TDOS of the model system of F6TCNNQ/ML‐MoS_2_/HOPG as a function of temperature. b) PDOS of the model system representing the partially occupied LUMO of F6TCNNQ as a function of temperature. Simulations were performed with occupation smearing (see text). The curves were all rigidly shifted by 5.4 eV, such that the electronic occupation distribution drops below 0.09 above 0 eV. The ratio of charged molecules (given in %) in the inset of (b) were calculated by integration of the local density of state of the LUMO orbital multiplied by the occupation distribution function (see the Experimental Section). The total area under the peaks in (b) integrate to two. The error bars correspond to the remaining statistical uncertainty of the stochastic sampling.

We use the total electronic DOS (TDOS) and the PDOS to analyze the possible impact of finite‐temperature nuclear motion on the CT of our vdW heterostructure, for the dilute case. We pick this structure because it is sufficient to grasp the CT, as shown in the previous section. The two aspects that can change the position of electronic states, namely thermal population of vibrational modes and thermal lattice expansion, are investigated separately here. In particular, thermal fluctuations are captured through thermodynamic averages on stochastically sampled nuclear configurations consistent with the distribution of quantum harmonic phonons at different temperatures^[^ ^32^, ^33^ ^]^ (details given in the Experimental Section and Sections S7 and S8 in the Supporting Information). The TDOS around the Fermi level in **Figure** **4**a (HSE06 functional) shows a pronounced shift to lower energies between 50 and 150 K. It also shows that the occupation of vibrationally excited states according to three different temperatures up to 300 K leads to an obvious broadening compared to the static calculation. The molecular partially occupied LUMO level dominates the TDOS within this energy window at all temperatures, as corroborated by the PDOS in Figure 4b. The area under this peak integrates to two, because there is no distinction between the two spin channels. Increasing the temperature results in a broadening of the partially occupied LUMO peak and a shift of its maximum toward lower energies (see Figure 4b). This also indicates an increase of occupation of this level, which can be interpreted as an increase of the fraction of molecular anions in the heterostructure. The electronic population of this state has been calculated through integration of the local PDOS with the electronic occupation distribution used in the calculations (see the Experimental Section) and is shown in the inset of Figure 4b. The fraction increases by around 30% from 50 to 300 K. Most of the changes are observed between 50 and 150 K, and the modes that become thermally populated in this temperature range correspond to hindered rotations of the molecules with respect to ML‐MoS_2_ and breathing modes of the MoS_2_ lattice, both of which influence the adsorption geometry.

The effect of thermal lattice expansion, which is comparably pronounced for MoS_2_,^[^ ^34^, ^35^ ^]^ was investigated with static calculations. The results shown in Figure S11 (Supporting Information) show that the effect of the expansion on the electronic orbitals is very small, but nevertheless shifts the partially occupied LUMO to lower energies with increasing temperature. Consequently, nuclear fluctuations and lattice expansion work hand in hand to promote CT in the vdW heterostructure with increasing temperature, as found in our experiment.

## 3 Discussion

Considering the above results for our specific vdW heterostructure consisting of the molecular electron acceptor F6TCNNQ, the 2D semiconductor MoS_2_, and the conductor HOPG, one reason for the temperature‐dependent amount of charge transfer can be rationalized as consequence of the electron–phonon coupling that is particularly pronounced for ML‐MoS_2_ and the molecules. The molecules’ LUMO level, which receives electrons from HOPG and slightly hybridizes with MoS_2_, is shifted to lower energies and broadened with increasing temperature, resulting in a larger fraction of molecules available for CT. In addition, it is well known that increasing temperature also induces a band‐gap renormalization for MoS_2_^[^ ^36^, ^37^ ^]^ (see Figures S12 and S13 in the Supporting Information). These phenomena, however, cannot be captured by the capacitor model discussed in the introduction, because the amount of CT is expected to depend on the DOS of the electron donor (HOPG) and acceptor (F6TCNNQ) only, and the energy levels of MoS_2_ in between should play no role. Hence, the electrostatic model neglects a further key aspect. As seen from our calculations, the finite electronic coupling between F6TCNNQ and MoS_2_, despite the weak vdW type of interaction, also influences the electronic ground state. The electronic coupling between the different parts of the heterostructure, and the temperature renormalization of the MoS_2_ energy gap and the molecular frontier orbitals, can also impact the character of the ground electronic state and thus the population of the molecular LUMO level. The semiconductor in the middle of the vdW heterostructure thus takes on an active role as “bridge” between electron donor and acceptor.

**Figure 5:**
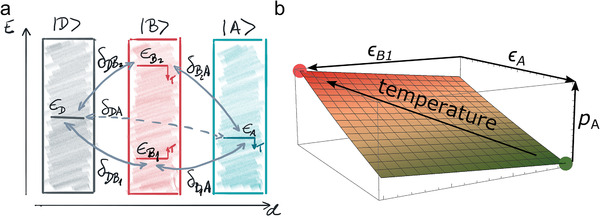
Sketch of simple single‐particle “donor–bridge–acceptor” model employed in this work. a) Relative energies (ε) of electronic levels of HOPG donor (D), MoS_2_ bridge (B), and F6TCNNQ acceptor (A) systems, schematically reflecting the isolated‐system level alignment (see Section S9 in the Supporting Information). Electronic couplings are denoted by δ. Effect of temperature (*T*) on the energy levels due to electron–phonon coupling is denoted by arrows. b) With increasing temperature, the relative position of ε changes due vibronic couplings. The population of the adiabatic state |*A*〉 (*p*_A_) in the ground state of the interacting system is sensitive to the renormalization of the molecular LUMO level (|*A*〉) and the VBM of MoS_2_ (|*B*_1_〉) as schematically shown in the figure.

A simple illustration of this effect can be achieved by a single‐particle “donor–bridge–acceptor” toy model. In this model, we denote the donor (D), bridge (B), and acceptor (A), with their respective energy levels ε_*j*_ (identified by corresponding subscripts), as well as the electronic couplings between all levels by *δ_nm_* (and corresponding subscripts), as schematically shown in **Figure** **5**a. The resulting Hamiltonian in the adiabatic basis of the isolated components (see details in Section S9 in the Supporting Information) can be constructed by the calculation of ε_*j*_ and by assuming reasonable values for the couplings in vdW heterostructures (0.1–0.5 eV) as discussed in more detail in the Section S9 in the Supporting Information. Solving the Schrödinger equation for this Hamiltonian shows that the ground state contains mainly contributions from the B_1_ and A states. The thermal population of state A (equivalent to molecular LUMO) in the ground state of the interacting system can be straightforwardly calculated. We can then analyze how this population changes by shifting the position of MoS_2_ and F6TCNNQ energy levels according to their temperature‐induced vibronic renormalization, which we have also accurately calculated for each component in isolation (see Section S8 in the Supporting Information). The qualitative picture sketched in Figure 5b is found, which changes only quantitatively with different values of couplings in the system. Going along the ε_A_‐axis would correspond to considering only a renormalization of the LUMO level of the molecule with temperature, while moving along the ε_B1_‐axis considers only the temperature‐dependent VBM renormalization of MoS_2_. To obtain the full effect of temperature on the ground‐state CT from D to A, however, necessitates moving along both directions simultaneously, as apparent from the plot. This coupled effect would be absent without the bridge. Although this model is too simple and empirical for a full understanding of the physical processes involved in the real system, it captures the key components that aid CT within vdW heterostructures.

## 4 Conclusions

The amount of ground‐state charge transfer to reach electronic equilibrium in multicomponent vdW heterostructures is found to be dependent on the intracomponent electron–phonon coupling and the intercomponent electronic coupling. Both couplings lead to a temperature dependence of the electronic levels, and therefore of the charge transfer. The change in the amount of transferred charge is due to a change of the density of charged versus neutral molecules, while a molecular ion carries one integer charge independent of temperature. Here, this is exemplified for a prototypical vdW heterostructure consisting of graphite/monolayer‐MoS_2_/F6TCNNQ, where the ground‐state charge transfer amount at room temperature is found to be ≈3 times higher than at 7 K. These findings are evidently significant for all studies and applications of vdW heterostructures where temperature is a variable. Furthermore, this insight could be used to bestow electronic and optoelectronic devices with unique temperature‐dependent functionalities.

## 5 Experimental Section

### Sample Preparation

ML‐MoS_2_ were grown on sapphire via chemical vapor deposition (CVD) and transferred onto an HOPG substrate by conventional poly(methyl methacrylate) (PMMA) transfer method.^[^ ^38^, ^39^, ^40^ ^]^ The quality of samples was evaluated before photoemission measurement using optical and atomic force microscopy (see Figures S2 and S3 in the Supporting Information). Sample cleaning step of 300–350 °C in situ annealing in preparation chamber (base pressure of 10^–10^ mbar) was done to get rid of unwanted carbon‐based contamination including residual PMMA. F6TCNNQ (Novaled), as molecular acceptor, was deposited onto the clean MoS_2_/HOPG in the same preparation chamber while monitoring the nominal mass‐thickness by using a quartz crystal microbalance.

### Photoemission Measurements

ARPES spectra were measured at the beamline BL7U (UVSOR, Japan), the IMS (MBS‐A1 lab system, Japan) and the Humboldt University (lab system, Germany) using a hemispherical electron analyzer with monochromatic light source of 21 eV and 9 eV, respectively. The determination of total resolution of 100 meV at 300 K were performed using clean Au (111) single crystal. The energy calibration with respect to Fermi level were carried out by measuring the electrically grounded clean Au (111) single crystal.

### Theoretical Modeling

The calculations presented here were based on density functional theory (DFT),^[^ ^41^, ^42^ ^]^ as implemented in the all‐electron, numeric atom‐centered electronic‐structure package FHI‐aims.^[^ ^43^, ^44^ ^]^ The Perdew–Burke–Ernzerhof (PBE) generalized gradient approximation^[^ ^45^ ^]^ functional was employed for the atomic geometry relaxation, while the hybrid Heyd–Scuseria–Ernzerhof (HSE06) was used for the electronic properties to grasp the correct charge transfer and level alignment.^[^ ^46^ ^]^ The “tight” basis sets were used for PBE calculations, while the “intermediate” settings were used for HSE06. All the calculations include the Tkatchenko–Scheffer van der Waals (TS–vdW) interactions^[^ ^47^ ^]^ and non‐self‐consistent spin–orbital coupling (SOC).^[^ ^48^ ^]^ Comparison of TS–vdW with the many body dispersion of ref. ^[^ ^49^ ^]^ shows that the corrugation of the potential energy surface is similarly described by these methods (see details in Table S2 in the Supporting Information). A vacuum region of 100 Å and a dipole correction were employed to avoid the spurious electrostatic interactions between periodic supercells.^[^ ^47^ ^]^ A 20 × 20 × 1 *k*‐point sampling was used for the (1 × 1) ML‐MoS_2_ (0001) and graphite (001) surface to attain the correct electronic properties. Four layers of graphite (001) surface were considered to guarantee the convergence of work function, which is also reported in a previous experiment.^[^ ^50^ ^]^ The atoms in the two bottom layers were fixed while other atoms are allowed to fully relax. The minimum force on atoms is guaranteed to be below 0.001 eV Å^−1^.

A grid search was performed initially to find the most stable geometry of one molecule adsorbed on free‐standing MoS_2_ with lying‐down and standing‐up geometries at different packing densities. At the low packing density under the supercell of (6 × 6), 41 models for lying‐down were simulated by PBE functionals, and 10 models were selected to perform the electronic property calculations. For the higher packing densities, 22 models were considered. This method is described in detail in the previous work.^[^ ^25^ ^]^ To simulate MoS_2_ (0001)/graphite (001), a typical commensurate superstructure with (4 × 4) MoS_2_ and (5 × 5) graphite was considered, to reduce the lattice mismatch. Based on this moiré superstructure, six models with one molecule lying‐down on the supercell graphite (5 × 10)/MoS_2_ (4 × 8) and three models with molecule lying‐down, short‐tilted, and long‐tilted on the supercell graphite (5 × 5)/MoS_2_ (4 × 4) were considered to perform the geometry optimization with the PBE functional. Then, a random structure search strategy^[^ ^51^ ^]^ was used to find geometries of two molecules lying flat at the (4 × 8) MoS_2_ supercells. The most stable structure was selected for consideration with the graphite substrate. In the theory part here, HOPG refers to a graphite (001) (5 × 10) supercell. All calculations have been performed with a Gaussian occupation smearing with a width of 150 meV to ensure convergence of the self‐consistent cycles. The (P)DOS shown in the manuscript have been shifted such that this occupation function assumes values below 0.09 above the zero of energy.

The Hessian matrix was constructed and diagonalized for the F6TCNNQ/ML‐MoS_2_ (4 × 8)/graphite (5 × 10) system by using the phonopy package,^[^ ^52^ ^]^ in which only atoms of F6TCNNQ and MoS_2_ were displaced by 0.01 Å, in the presence of the graphite substrate. Here, forces were calculated with the PBE+TS–vdW functional in the supercell. The phonon modes obtained in this way were used to create the thermal displacements as detailed in Section S8 in the Supporting Information. Then, 35, 50, and 35 configurations were used to calculate TDOS and PDOS of the F6TCNNQ/ML‐MoS_2_ (4 × 8)/graphite (5 × 10) [522 atoms] at 50, 150, and 300 K, respectively.

## Conflict of Interest

The authors declare no conflict of interest.

## Author Contributions

S.P. and H.W. contributed equally to this work. S.P., N.K., and M.R. conceived and supervised the project. S.P., T.S., M.M., T.Y., Y. H., P.A., and S.K. performed ARPES measurements and analyzed the data, under supervision of N.K. A.A., A.H., L.L., S.K., and V.C.T. prepared ML‐MoS_2_ samples. H.W., M.Z., D.M., and M.R. performed all calculations and their analysis. S.P., H.W., N.K., and M.R. prepared the manuscript. All authors commented on the manuscript.

## Supporting information

Supporting Information

## Data Availability

The data that support the findings of this study are available from the corresponding author upon reasonable request.
