# Supplementary material for: Temperature‐Dependent Electronic Ground‐State Charge Transfer in van der Waals Heterostructures
Source: Adv Mater. 2021 May 25;33(29):2008677. doi: 10.1002/adma.202008677 (PMC11468622; doi:10.1002/adma.202008677)
Supplement: Supplementary file 1 — Supporting Information [file ADMA-33-2008677-s001.pdf]

© 2021 Wiley-VCH GmbH

ADVANCED MATERIALS

Supporting Information

for *Adv. Mater.,* DOI: 10.1002/adma.202008677

Temperature-Dependent Electronic Ground-State Charge Transfer in van der Waals Heterostructures

Soohyung Park, Haiyuan Wang, Thorsten Schultz, Dongguen Shin, Ruslan Ovsyannikov, Marios Zacharias, Dmitrii Maksimov, Matthias Meissner, Yuri Hasegawa, Takuma Yamaguchi, Satoshi Kera, Areej Aljarb, Mariam Hakami, Lain-Jong Li, Vincent Tung, Patrick Amsalem, Mariana Rossi, and Norbert Koch**

Supplementary Information for

Temperature-dependent electronic ground state charge transfer in van der Waals heterostructures

Soohyung Park,[1,†] Haiyuan Wang[2,3,†], Thorsten Schultz,[4,5] Dongguen Shin,[4] Ruslan Ovsyannikov,[5] Marios Zacharias,[2,6] Dmitrii Maksimov,[2,7] Matthias Meissner,[8] Yuri Hasegawa,[8] Takuma Yamaguchi,[8] Satoshi Kera,[8] Areej Aljarb,[9] Mariam Hakami,[9] Lain-Jong Li,[9,10] Vincent Tung,[9] Patrick Amsalem,[4] Mariana Rossi,[2,7,*] and Norbert Koch[4,5,*]

[1] Advanced Analysis Center, Korea Institute of Science and Technology (KIST), Seoul 02792, South Korea

[2] Fritz Haber Institute of the Max Planck Society, 14195 Berlin, Germany

[3] Chaire de simulation à l'échelle atomique (CSEA), Ecole Polytechnique Fédérale de Lausanne (EPFL), CH-1015 Lausanne, Switzerland

[4] Humboldt-Universität zu Berlin, Institut für Physik & IRIS Adlershof, 12489 Berlin, Germany

[5] Helmholtz-Zentrum für Materialien und Energie GmbH, 12489 Berlin, Germany

[6] Department of Mechanical and Materials Science Engineering, Cyprus University of Technology, 3603 Limassol, Cyprus

[7] Max Planck Institute for the Structure and Dynamics of Matter, 22761 Hamburg, Germany

[8] Institute for Molecular Science, 444-8787 Okazaki, Japan

[9] Physical Sciences and Engineering, King Abdullah University of Science and Technology, Thuwal 23955-6900, Saudi Arabia

[10] Department of Mechanical Engineering, The University of Hong Kong, Pok Fu Lam Road, Hong Kong

[†] both authors contributed equally to this work

* corresponding authors: rossi@fhi-berlin.mpg.de, norbert.koch@physik.hu-berlin.de

Table of contents

Section 1. Temperature-independent charge transfer in F6TCNNQ/HOPG

Supplementary Figure S1 | F6TCNNQ on HOPG at different temperatures.

Section 2. Quality of ML-MoS_2/HOPG

Supplementary Figure S2 | Optical microscopy (OM) image of ML-MoS_2/HOPG

Supplementary Figure S3 | Atomic force microscopy (AFM) image of ML-MoS_2/HOPG

Section 3. Estimation of the fraction of charged F6TCNNQ at 300 K.

Supplementary Figure S4 | UPS measurement of secondary electron cutoff region of ML-MoS_2 with/without F6TCNNQ

Supplementary Table S1 | Impact of temperature on the amount of charge transfer and the fraction of charged molecule that estimated in a proportional way using ξ from 300 K.

Section 4. ARPES spectra of ML-MoS_2 by molecular acceptor with temperature

Supplementary Figure S5 | Energy distribution curve (EDC) of charged F6TCNNQ as a function of temperature.

Supplementary Figure S6 | Angle resolved photoelectron spectroscopy (ARPES) spectra of ML-MoS_2 by molecular doping.

Section 5. Determination of orientation of F6TCNNQ on top of ML-MoS_2 with temperature

Supplementary Figure S7 | Temperature dependent x-ray absorption spectroscopy (XAS).

Section 6. Calculated properties of F6TCNNQ on the free-standing ML-MoS_2

Supplementary Figure S8 | The atomic structure of F6TCNNQ on ML-MoS_2

Supplementary Figure S9 | Calculated electronic band structure and projected density of states.

Supplementary Figure S10 | Visualization of electronic orbitals in F6TCNNQ/ML-MoS_2/HOPG.

Supplementary Table S2 | Impact of vdW interactions.

Section 7. Temperature dependence of electronic levels

Supplementary Figure S11 | The PDOS of F6TCNNQ in the system considering only lattice expansion at different temperatures.

Section 8. Stochastic sampling of the vibrational space

Supplementary Figure S12 | The effect of thermal displacement on DOS of ML-MoS_2.

Supplementary Figure S13 | Projected electronic density of states of MoS_2 and HOPG at the static and at 150 K for the F6TCNNQ/ML-MoS_2/HOPG.

Supplementary Table S3 | VBM and CBM renormalization by thermal displacement of ML-MoS_2.

Supplementary Table S4 | HOMO and LUMO renormalization by thermal displacement of F6TCNNQ.

Supplementary Table S5 | Summarized energy level donor-bridge-acceptor at 0 K.

Section 9. Minimal model to understand T dependence of level occupations

Supplementary Figure S14 | Population of the molecular level upon variation of the relative VBM and LUMO energies.

Section 1. Temperature-independent charge transfer in F6TCNNQ/HOPG

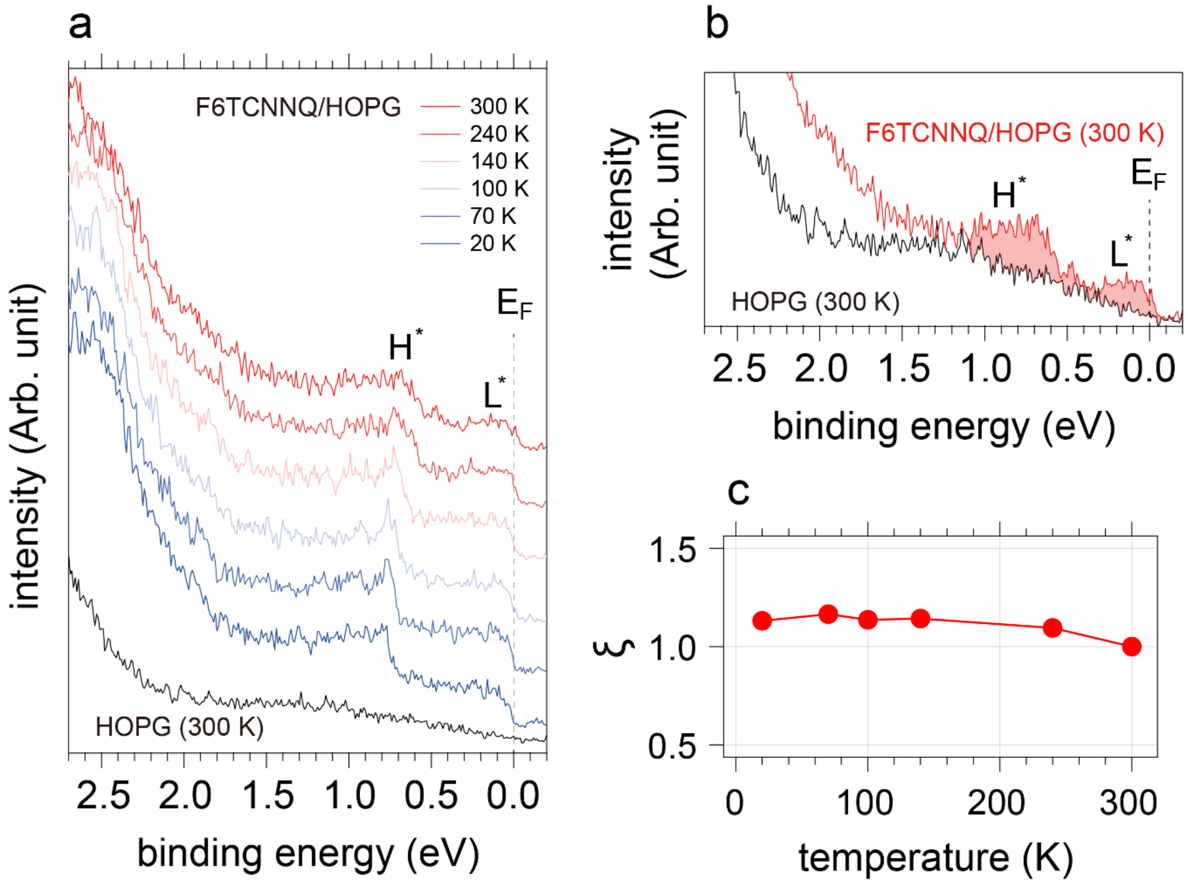

Supplementary Figure S1 | F6TCNNQ on HOPG at different temperatures. (a) Energy distribution curves (EDC) of 0.5 monolayer (ML) F6TCNNQ on HOPG and clean HOPG near the Fermi-level as a function of temperature around K point (angle integrated for 22°) by using He I_{α}. (b) EDCs of 0.5 ML F6TCNNQ/HOPG and clean HOPG spectra at 300 K. L^* and H^* denote the partially filled LUMO and relaxed HOMO of F6TCNNQ representing the charged F6TCNNQ. Colored area indicating the charged F6TCNNQ features, which are summarized in Figure c. (c) Relative change in charged F6TCNNQ (ξ) as a function of temperature, which shows that amount of charged F6TCNNQ is not significantly changed by temperature.

To understand the impact of temperature on the charge transfer between F6TCNNQ and HOPG alone, ARPES measurement were carried out as shown in Figure S3 and it shows only a sharpening of the F6TCNNQ features without a change of spectral weight (area). This shows that the ratio of neutral and charged F6TCNNQ molecules is not influenced by temperature.

Section 2. Quality of ML-MoS_2/HOPG

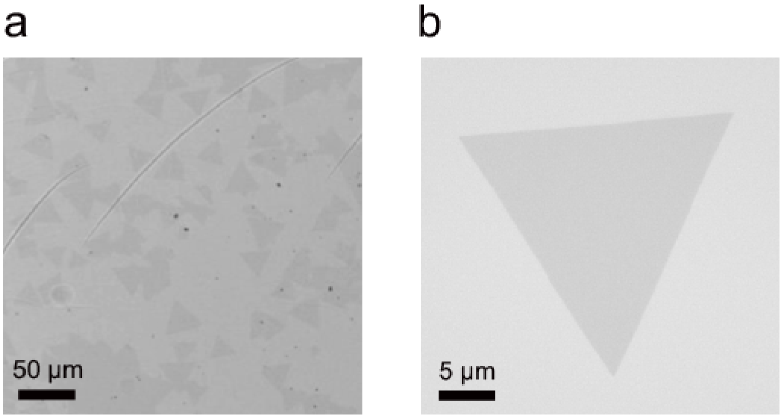

Supplementary Figure S2 | Optical microscopy images of ML-MoS_2/HOPG. (a) Low magnification image from the sample edge with about 50 % coverage of ML-MoS_2 on top of HOPG; the sample center (where ARPES data were obtained) has an average ML-MoS2 coverage above 80%. (b) Zoon-in image showing a sharp and equilateral ML-MoS_2 triangle.

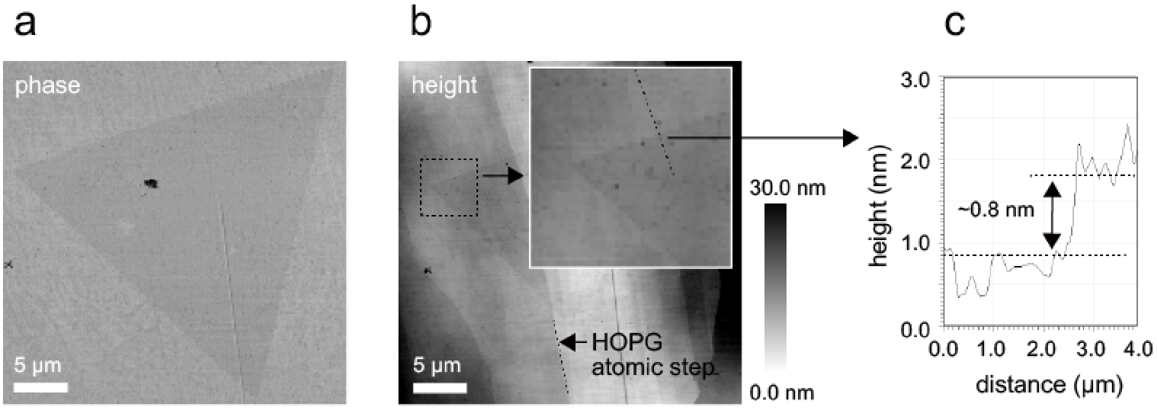

Supplementary Figure S3 | Atomic force microscopy (AFM) images of ML-MoS_2/HOPG. (a) Phase and (b) height AFM image of ML-MoS_2. (c) Height profile measured along the dashed line in the inset of (b) indicates ML-height (~ 0.8 nm).

Section 3. Estimation of the fraction of charged F6TCNNQ at 300 K.

To quantitatively estimate the amount of charge transfer, the fraction of charged F6TCNNQ molecules is calculated from the transferred electron density divided by the density of F6TCNNQ on top of the monolayer (ML) MoS_2 surface ($\rho_e/\rho_{F6TCNNQ}$), based on the assumption that one electron can be transferred into only one molecule.

(1) Density of F6TCNNQ on top of ML-MoS_2 surface

In the F6TCNNQ deposition step, the nominal thickness was monitored by a quartz crystal microbalance (QCM), which enables us to calculate the density of F6TCNNQ on top of ML-MoS_2 using the following equation:

$$\rho_{\mathrm{F6TCNNQ}} = \frac{N_{\mathrm{F6TCNNQ}}}{A} = \frac{S}{\mu} d$$

where ρ_{F6TCNNQ}, N_{F6TCNNQ}, A, S, μ and d are the density of F6TCNNQ on top of ML-MoS_2 surface, the number of F6TCNNQ molecules, area, mass density used in monitoring the nominal thickness by QCM, molecular molar mass, and nominal thickness taken from the QCM, respectively. The S of 1.3 g/cm^3, d of 5Å (~0.5 ML), and μ of 362.2 g/mol were used, and the estimated ρ_{F6TCNNQ} of 1.08×10^{14} molecule/cm^2 was obtained.

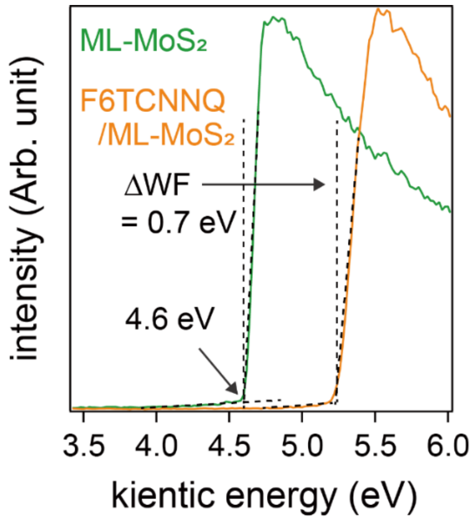

Supplementary Figure S4 | UPS measurement of secondary electron cutoff region of ML-MoS_2 with/without F6TCNNQ at 300 K. The spectrum shift of 0.7 eV shows the change in work function due to charge transfer from HOPG to F6TCNNQ.

(2) Transferred electron density

The density of transferred charges can be calculated using the Helmholtz equation as follows[1]:

$$\rho_e = \frac{\Delta \mathrm{WF} \cdot \varepsilon_{\mathrm{eff}} \varepsilon_0}{e d_{\mathrm{eff}}}$$

where $\Delta\mathrm{WF}, \varepsilon_0, \varepsilon_{\mathrm{eff}}, d_{\mathrm{eff}}$, and e are the work function change, the vacuum permittivity, the effective dielectric constant, the effective dipole distance (HOPG to F6TCNNQ), and the elementary charge, respectively. The parameters $\varepsilon_{\mathrm{eff}}$ = 5.46 (ML-MoS_2 for 6.4, F6TCNNQ for 3, and HOPG for 7), d_{eff} = 9 Å, and the measured ΔWF of 0.70 eV were used. As a result, the transferred charge density ρ_e at 300 K was calculated to be $2.35\times10^{13}/\mathrm{cm}^2$.

From that, we now can estimate the fraction of charged F6TCNNQ at 300 K to be 21.71 % ($\rho_e/\rho_{F6TCNNQ}$), and that at 7 K to be 7.24 % in the proportional way using ξ at 7 K as defined in the main manuscript text ($\xi_{@7K} \cdot \rho_e/\rho_{F6TCNNQ}$).

Supplementary Table S1 | Impact of temperature on the amount of charge transfer and the fraction of charged molecules, obtained according to the procedure described above.

**temperature (K)**	**300**	**240**	**200**	**140**	**100**	**70**	**7**
**amount of charge transfer ($\times10^{13}/\mathrm{cm}^2$)**	2.35	2.26	2.35	2.09	1.76	1.18	0.84
**fraction of charged F6TCNNQ molecules (%)**	21.7	20.8	21.7	19.3	16.3	10.9	7.24

Section 4. ARPES spectra of ML-MoS_2 + molecular acceptor for different temperatures

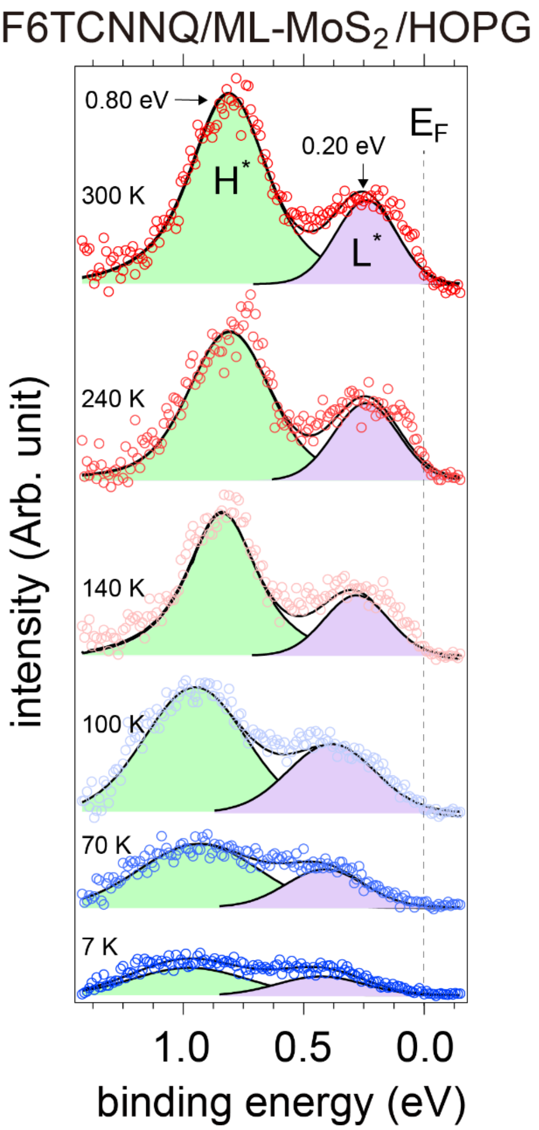

Supplementary Figure S5 | Energy distribution curve (EDC) of charged F6TCNNQ as a function of temperature. To obtain the reliable binding energy of filled LUMO (L*) and relaxed HOMO (H*), the HOPG substrate feature were subtracted. The energetic position difference and intensity ratio between H* and L* were fixed and checked from previous work[1] during the fitting process.

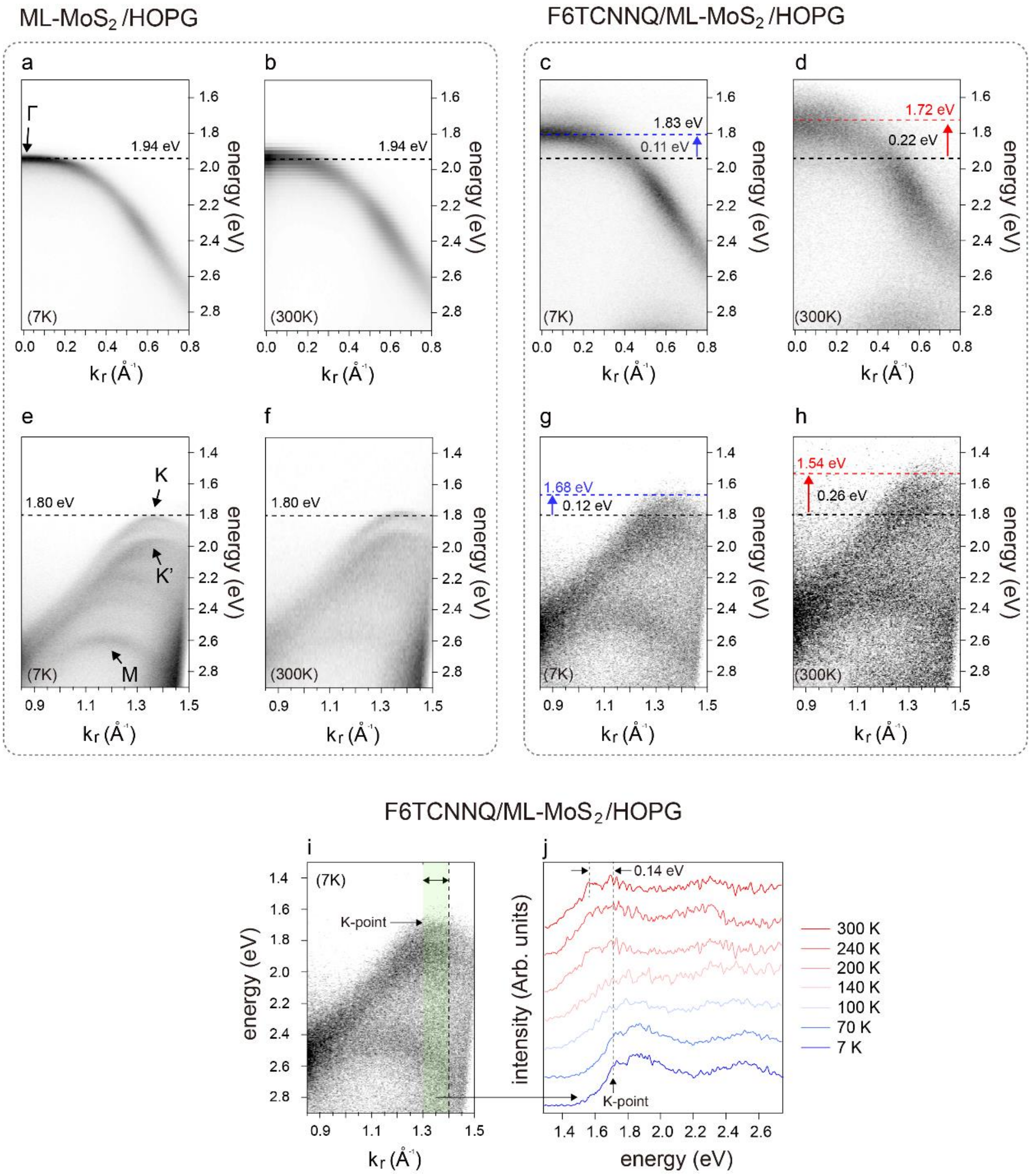

Supplementary Figure S6 | Angle resolved photoelectron spectroscopy (ARPES) spectra of ML-MoS_2 by molecular doping. ML-MoS_2 /highly oriented pyrolytic graphite (HOPG) ARPES spectra were measured around Γ (a-d) and K (e-h) point of the Brillouin zone (BZ) along the Γ to K+M direction. The spectra were collected at 7 K (a, c, e and g) and 300 K (b, d, f and h) with monochromatic photon energy of 21 eV. (c), (d), (g) (h) and (i) corresponding ARPES spectra after deposition of nominally 0.5 ML of F6TCNNQ. (j) Energy distribution curves obtained by integration of Fig. (i) around the K point in a narrow momentum interval [1.30-1.40 $Å^{-1}$] for varying temperature, showing the spectral shift towards lower energy for lower temperature.

In order to address the effect of temperature on the band structure of ML-MoS_2 without F6TCNNQ, ARPES spectra of clean ML-MoS_2/HOPG were measured with increasing temperature from 7 K to 300 K in Figure S6 left dashed line frame. Figure S6a and b show that ARPES spectra of ML-MoS_2 around the Γ point of BZ for the selected temperatures. The local valence band maximum (VBM) at Γ point is estimated by 1.94 eV, which is in good agreement with previous reported values.[1,2] In Figure S6e and f, we found two split bands with local valley (K-K') around K point due to the lack of inversion symmetry and spin-orbit coupling.[3,4] The overlap of the bands along the two high symmetry directions Γ-M and Γ-K is an intrinsic feature of 2H phase ML-MoS_2 in azimuthally disordered sample.[5]

It is noteworthy that the spectra of the two extreme temperatures (7 K, 300 K) are nearly identical except for the spectral broadening originating from thermal fluctuation of the atoms. From that, it can be concluded that the band structure of ML-MoS_2 itself does not change by thermal fluctuations within the experimental resolution.

Upon deposition of a nominal 0.5 ML of F6TCNNQ, the band structure of ML-MoS_2 moves closer to the Fermi level by ca. 0.11 eV at both Γ and K points of BZ at 7 K as shown in Figure S6c and g. With increasing temperature up to 300 K, the band structure of ML-MoS_2 is gradually shifted towards the Fermi level resulting in a lower binding energy of VBM of 1.72 eV at Γ and 1.54 eV at K point, respectively. Therefore, all measured spectra show a gradual and rigid shift toward the Fermi level in the whole BZ upon stepwise increase of the temperature as summarized in Figure 2c in main manuscript. In addition, we also found a significant broadening of spectra originating from inelastic scattering of emitted photoelectrons from ML-MoS_2 by the adsorbed molecules.

In short, we did not find any evidence for any change in the electronic structure of ML-MoS_2 itself as a function of temperature. On the other hand, it is observed that the amount of ML-MoS_2 energy shift when molecules are adsorbed depends notably on temperature.

Section 5. Determination of orientation of F6TCNNQ on top of ML-MoS_2 with temperature

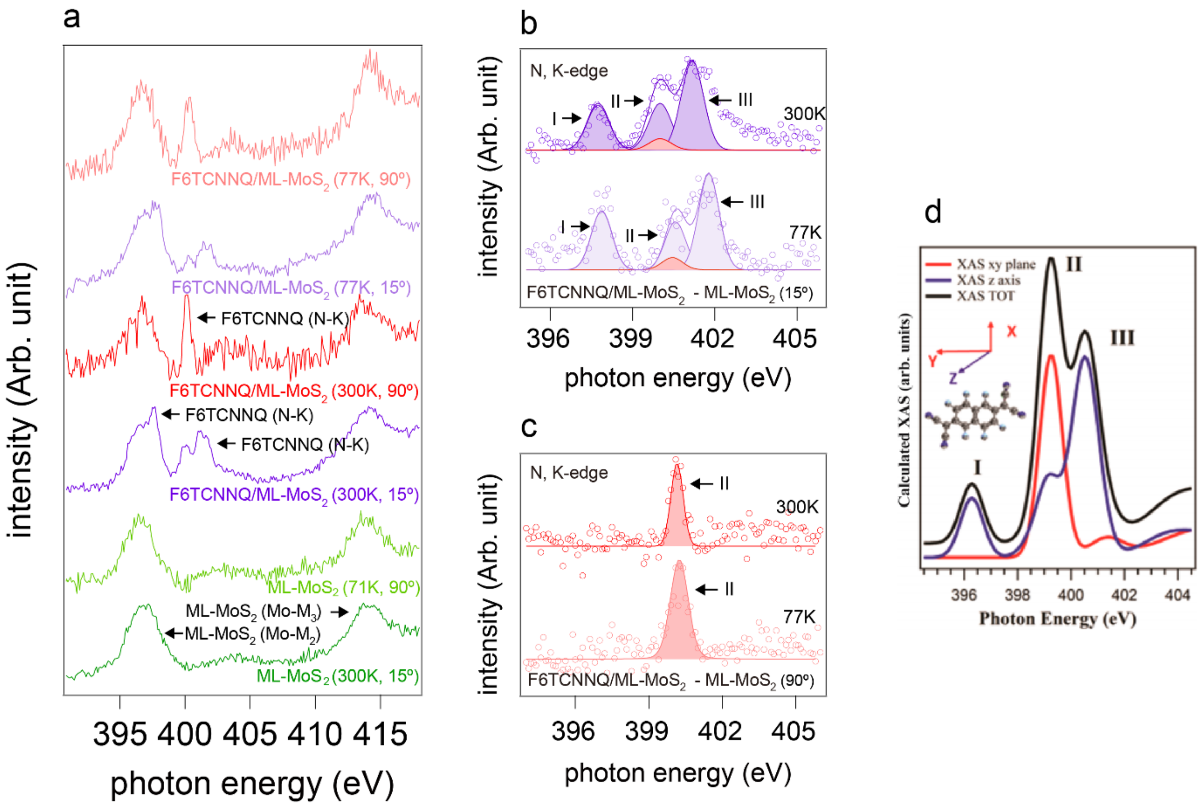

Supplementary Figure S7 | Temperature dependent x-ray absorption spectroscopy (XAS). (a) Selected XAS spectra of ML-MoS_2/HOPG and F6TCNNQ (0.5 ML)/ML-MoS_2/HOPG by varying the temperature and the angle to surface. The ML-MoS_2 spectra of other conditions are not shown here as they did not exhibit significant changes within the experimental resolution. The arrows denote the XAS edge for Mo M_2-edge, M_3-edge, and N K-edge, respectively. The ML-MoS_2/HOPG-subtracted XAS spectra of F6TCNNQ (0.5 ML)/ML-MoS_2/HOPG recorded at (b) 15° and (c) 90° varying the temperature. (d) Simulated *xy* plane (red) and *z*-axis (blue) N K-edge XAS spectra of F6TCNNQ. The xy plane and z-axis spectra denote that the absorption in the xy plane of F6TCNNQ and axis perpendicular to F6TCNNQ, respectively. XAS TOT (black) is the simulated total absorption. Reprinted with permission from Ref. 6. Copyright (2015) American Chemical Society

In order to determine the orientation of F6TCNNQ on top of HOPG at different temperatures, x-ray absorption spectroscopy was performed at the beamline PM4 (Bessy II, Germany). First, ML-MoS_2 /HOPG was annealed at 300°C in the preparation-chamber to obtain a clean surface. The XAS spectra of clean ML-MoS_2 /HOPG in N-K edge energy range were measured by varying the temperature from 300 K to 71 K and the angle between incident beam and surface normal from 15° to 90°, respectively. The spectra of ML-MoS_2/HOPG measured at different angles and temperatures are identical, therefore, only two selected extreme cases are shown in Figure S7 bottom two spectra. Since there

were no nitrogen atoms present, only the Mo-M_2 and M_3 edge features originating from ML-MoS_2 are observed without temperature and angle dependency. This enables to subtract the overlapping of ML-MoS_2/HOPG features easily from the spectra of F6TCNNQ/ML-MoS_2/HOPG as shown in Figure S7b and explained below.

As shown in the middle two spectra in Figure S7, varying the angle between the sample and incident light significantly changes the N-K edge feature coming from the F6TCNNQ. On the other hand, the change of temperature does not impact the N-K edge feature except for its sharpness. This is a clear evidence that F6TCNNQ on ML-MoS_2 adopts the same orientation with respect to the surface normal regardless of temperature.

As plotted in Figure S7b and c, we then subtract the contribution from ML-MoS_2 from the XAS spectra of F6TCNNQ/ML-MoS_2/HOPG in order to further analyze the N-K edge of F6TCNNQ. In Figure S7b and c, XAS spectra are further deconvoluted by one red and three violet Gaussians, according to previous reports as shown in Figure S7d (xy-plane for red peak and z-axis consisting of three blue peaks), respectively.[6] From this assignment, the orientation of F6TCNNQ is found to be lying on top of ML-MoS_2 surface regardless of the temperature.

Section 6. Calculated properties of F6TCNNQ on the free-standing ML-MoS_2

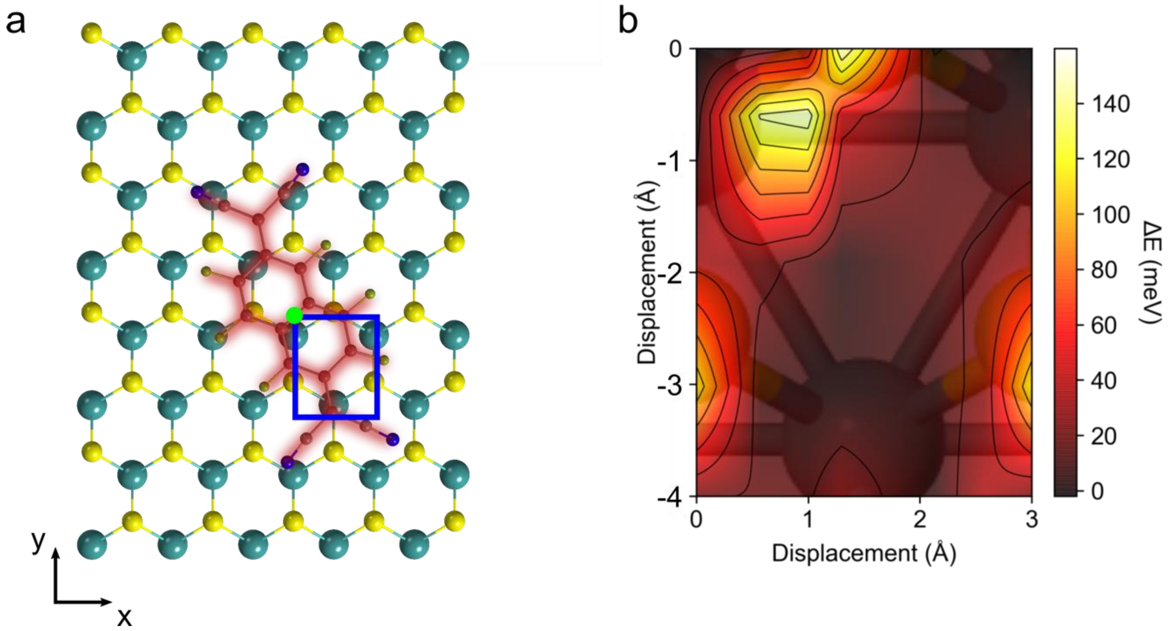

Supplementary Figure S8 | The atomic structure of F6TCNNQ on ML-MoS_2 (a) Lowest energy structure of F6TCNNQ on ML-MoS_2 as obtained with the HSE06+TS-vdW functional. (b) Corrugation of the potential energy landscape at different adsorption sites of F6TCNNQ on ML-MoS_2. The energy landscape was obtained by shifting the centre of mass of the molecule, constraining the central 2 carbon atoms in the x and y direction and relaxing all other degrees of freedom. The axis denotes the position marked by the green point in Figure a, and the area in the blue box corresponds to Figure b.

Supplementary Table S2 | Impact of vdW interactions. For four adsorption structure models randomly selected from the free standing F6TCNNQ/ML-MoS_2 structures and the most stable F6TCNNQ/ML-MoS_2/HOPG model MBD-nl calculations were performed. The adsorption energy per molecule is defined as $E_{\mathrm{ads}} = \frac{1}{n}\left(E^{\mathrm{total}}_{\mathrm{F6TCNNQ/MoS_2/HOPG}} - E^{\mathrm{total}}_{\mathrm{F6TCNNQ}} - E^{\mathrm{total}}_{\mathrm{MoS_2/HOPG}}\right)$, where n is the number of molecule, and E is the total energy of different slabs.) All the results based on fully relaxed geometries from HSE06+TS here. TS overestimates the adsorption energy but predicts a similar corrugation of the energy landscape as MBD-nl (see energy difference between different models).

		E_{ads} (eV)	
		TS	MBD_nl
F6TCNNQ/ML-MoS_2	Model 1	-1.764	-1.101
	Model 2	-1.825	-1.156
	Model 3	-1.849	-1.166
	Model 4	-1.828	-1.144
F6TCNNQ/ML-MoS_2/HOPG		-1.514	-0.773

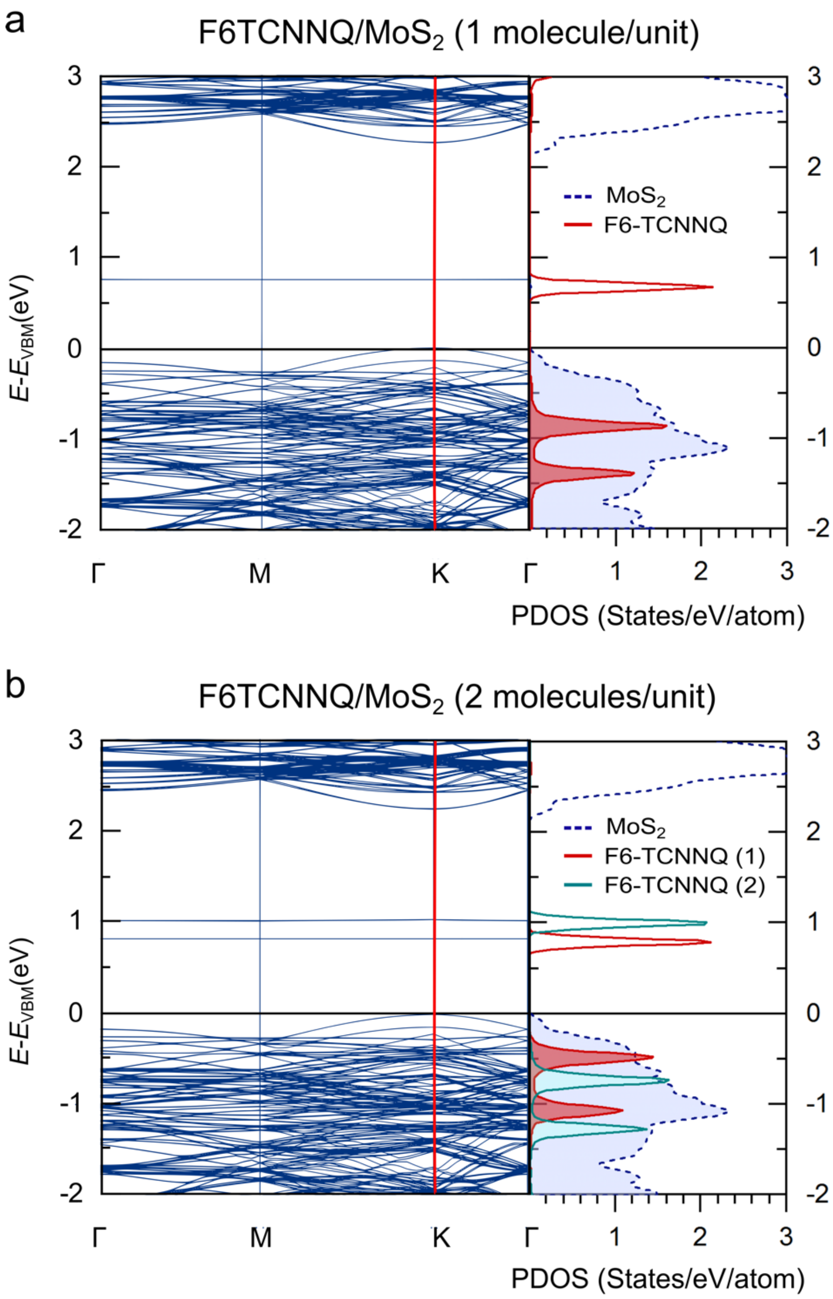

Supplementary Figure S9 | Calculated electronic band structure and projected density of states. Calculated band structure and projected density of states for dilute (a) and ML (b) F6TCNNQ on free-standing ML-MoS_2 (4×8 supercell) with the HSE06 functional. Band structure is folded.

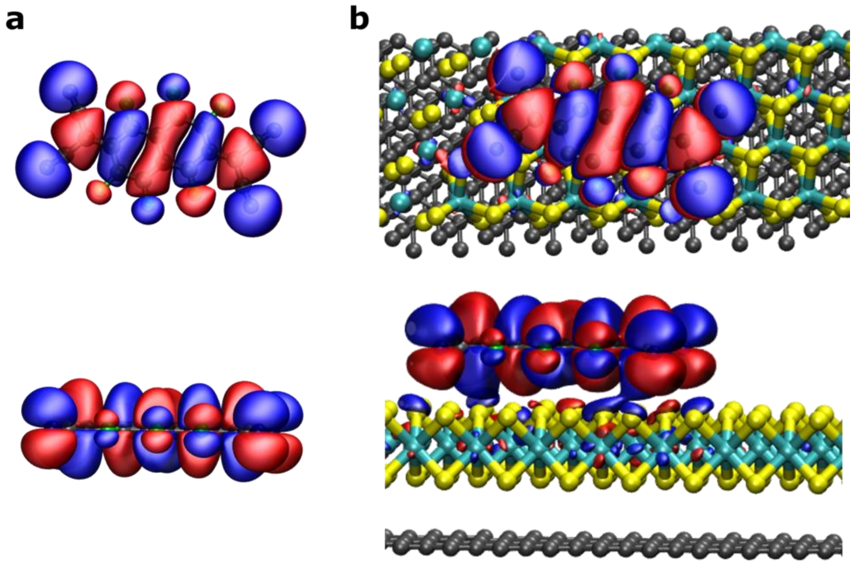

Supplementary Figure S10 | Visualization of electronic orbitals in F6TCNNQ/ML-MoS_2/HOPG. Visualization of (a) the LUMO of the isolated F6TCNNQ molecule and (b) of the partially filled LUMO in the F6TCNNQ/ML-MoS_2/HOPG system. Isosurfaces of +/- 0.01 e/Å$^{3/2}$ are shown.

Section 7. Temperature dependence of electronic levels

We here address temperature-dependence, caused by electron-phonon coupling, of the electronic density of states obtained from the single-particle Kohn-Sham levels $\varepsilon_{n,k}$ calculated with the HSE06 functional. Following the discussion in Ref. 7, we separate this temperature dependence as follows. Assume that a variation in the electronic density of states $D(E)$ as a function of volume V and temperature T is given by

$$dD(E,V,T) = \left(\frac{\partial D}{\partial T}\right)_V dT + \left(\frac{\partial D}{\partial V}\right)_T dV. \qquad (1)$$

Then we can write that its variation with temperature at a constant pressure is

$$\left(\frac{\partial D}{\partial T}\right)_P = \left(\frac{\partial D}{\partial T}\right)_V + V\left(\frac{\partial D}{\partial V}\right)_T \frac{1}{V}\left(\frac{\partial V}{\partial T}\right)_P = \left(\frac{\partial D}{\partial T}\right)_V + \left(\frac{\partial D}{\partial \ln V}\right)_T \left(\frac{\partial \ln V}{\partial T}\right)_P. \qquad (2)$$

In Eq. 2, we identify that the full temperature dependence is composed by the variation of D with temperature at a fixed volume plus its variation with volume at a fixed temperature, multiplied by the thermal expansion coefficient $\alpha = \left(\frac{\partial \ln V}{\partial T}\right)_P$.

We address the last term of Eq. 2 by taking the experimental thermal expansion coefficients for graphite and ML-MoS_2 from references[8,9] and scaling the ground-state lattice constants by this factor at the temperatures of 50, 150 and 300 K. Since there is a mismatch in the in-plane expansion coefficient of graphite and ML-MoS_2, we approximated these lattice constants by their predicted

average at each temperature, in order to minimize strain. We then calculated the ground-state $D(E)$ at each volume, consistent with T = 50, 150, 300 K, which is shown in Figure S11. The first term of Eq. 2 describing the electron-phonon effect on D, is more challenging to address, and we detail the procedure in the following section.

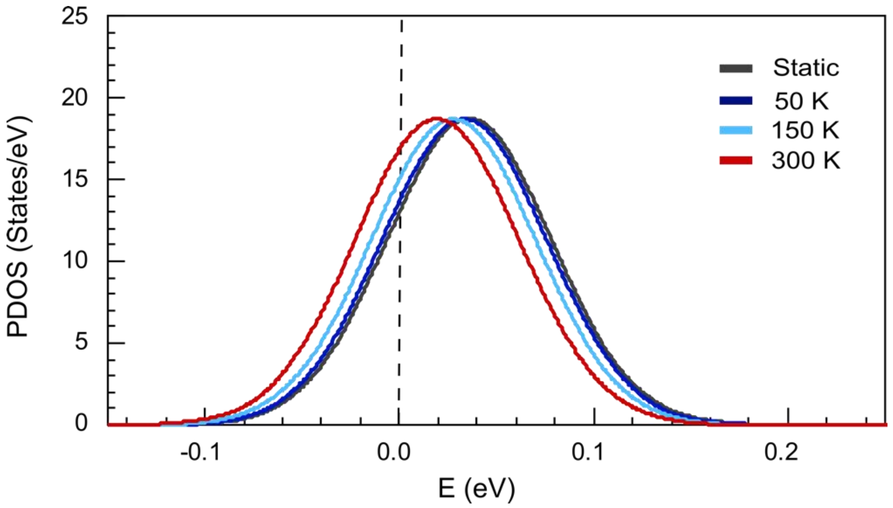

Supplementary Figure S11 | The PDOS of F6TCNNQ in the system considering only lattice expansion at different temperatures. Projected density of states has been consistently shifted by 5.4 eV, see main text.

Section 8. Stochastic sampling of the vibrational space

In the framework of the Williams-Lax theory[10,11] and harmonic approximation, the temperature dependence of the electronic density of states D is obtained as a multidimensional Gaussian integral[12,13]:

$$D(E,T) = \prod_\nu \int dQ_\nu \frac{e^{-Q_\nu{}^2/2\sigma_{\nu,T}{}^2}}{\sqrt{2\pi}\sigma_{\nu,T}} D(E,Q), \quad (3)$$

where the product runs over all modes ν, and Q is used to indicate the configuration defined by the normal coordinates $\{Q_\nu\}$. D evaluated at the configuration Q is given by:

$$D(E,Q) = \sum_{n\boldsymbol{k}} \omega_{\boldsymbol{k}} \delta\big(E - \varepsilon_{n\boldsymbol{k}}(Q)\big), \quad (4)$$

where the summation is taken over all bands n and wave vectors $\boldsymbol{k}$ with weights $\omega_{\boldsymbol{k}}$, and $\varepsilon_{n\boldsymbol{k}}(Q)$ represents the Kohn-Sham energies of the system evaluated at configuration Q. Equation 3 represents precisely the thermal average of D at the fixed volume, and can be numerically evaluated using importance sampling Monte Carlo integration. This corresponds to (i) generating a variety of atomic configurations Q, at the fixed volume, from the distribution $\Pi_\nu e^{-Q_\nu{}^2/2\sigma_{\nu,T}{}^2}/\sqrt{2\pi}\sigma_{\nu,T}$, (ii) calculating the $D(E,Q)$ for each configuration, and (iii) taking the average of all calculated $D(E,Q)$. Regarding step (i), the widths of the multivariate Gaussian distribution are defined by the mean square displacement of the atoms along mode ν as[14]:

$$\sigma_{\nu,T} = l_\nu \sqrt{2n_{\nu,T} + 1}, \quad (5)$$

where $l_\nu = \sqrt{\hbar/2M_p\omega_\nu}$ is the zero-point vibrational amplitude, and $n_{\nu,T} = [e^{\frac{\hbar\omega_\nu}{k_B T}} - 1]^{-1}$ is the Bose-Einstein distribution. Additionally, M_p and ω_ν denote the mass of proton and the frequency of the νth normal mode. Taking Eq. 5 together with the set of frequencies and eigenmodes obtained with phonopy,[15] we can create a list of atomic displacements at a given temperature. In the following, the method of Sobol low-discrepancy sequences[16] was used to sample efficiently the normal coordinates.[12] Finally, 35, 50 and 35 configurations were used to calculate the electronic density of states (EDOS) of the F6TCNNQ/ML-MoS_2(4×8)/graphite (5×10) [522 atoms] for temperatures 50 K, 150 K, and 300 K, respectively. This amount of sampling ensured sufficient statistical convergence for the effects discussed in this manuscript. In the same way with the density of sates in the Eq. 3, we also calculated the temperature-dependent band gap for the free-standing ML-MoS_2 presented in Figure S12 and Table S3. The band gap becomes smaller as the temperature increases, with VBM and CBM being shifted up and down in energy, respectively, and the shift we observe agrees with the literature.[13,17] We have also calculated the renormalization of the levels of the F6TCNNQ molecule as shown in Table S4.

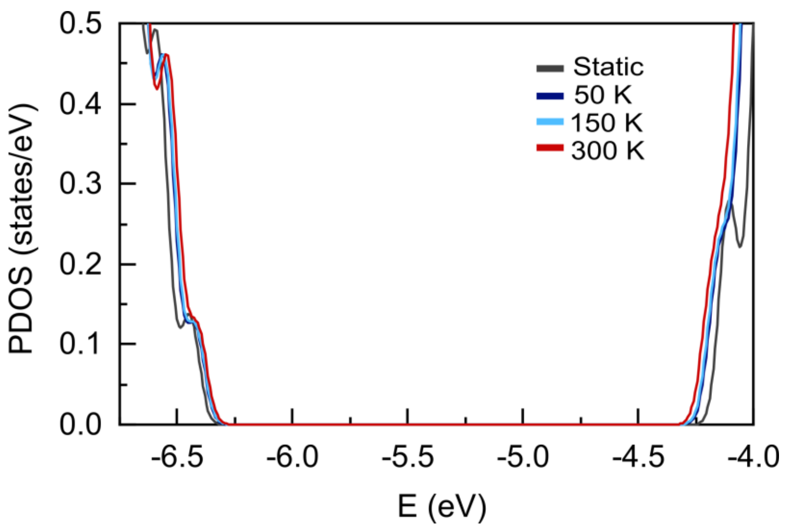

Supplementary Figure S12 | The effect of thermal displacement on DOS of ML-MoS_2. The DOS of ML-MoS_2 at different temperatures.

To complement the results shown for the full heterostructure in the main manuscript, in Figures S13(a) and (b) we also show the PDOS of MoS_2 and HOPG at 150 K. The hybridization of the MoS_2 states with the molecular states is clear. The band gap renormalization of MoS_2 is also confirmed, and the depopulation of HOPG states can be observed.

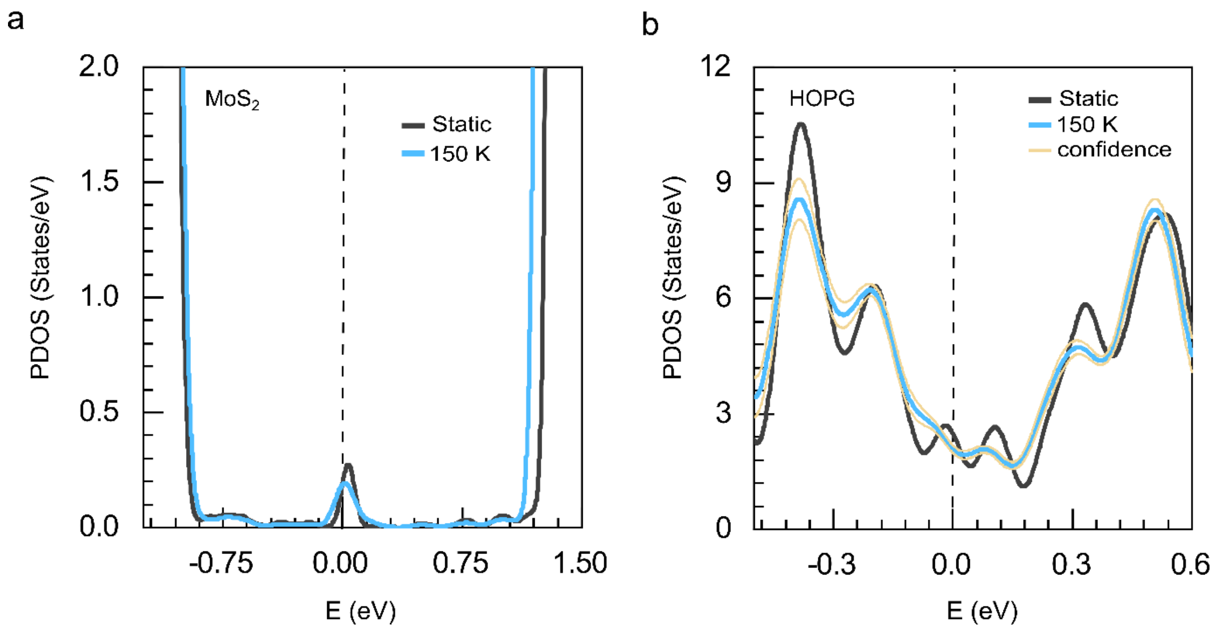

Supplementary Figure S13 | Projected electronic density of states of MoS_2 and HOPG for the static case and at 150 K for F6TCNNQ/ML-MoS_2(4×8)/graphite [522 atoms] (HSE06 functional). The curves were all rigidly shifted by 5.4 eV, see main text.

Supplementary Table S3 | VBM and CBM renormalization by thermal displacement of ML-MoS_2. Position of VBM and CBM of ML-MoS_2 as a function of temperature corresponding to Figure S12 (HSE06 functional, ZORA, SOC). In parenthesis, relative difference to static, in meV.

Temperature (K)	VBM (eV)	CBM (eV)	Band gap (eV)
**Static (0 K)**	-6.415	-4.075	2.340
**50 K**	-6.394	-4.117	2.277
**150 K**	-6.391	-4.123	2.268
**300 K**	-6.381 (+34)	-4.143 (-68)	2.238 (-102)

Supplementary Table S4 | HOMO and LUMO renormalization by thermal displacement of F6TCNNQ. Position of HOMO and LUMO of isolated F6TCNNQ as a function of temperature (HSE06 functional, ZORA, SOC). In parenthesis, relative difference to static, in meV.

Temperature (K)	HOMO (eV)	LUMO (eV)	Band gap (eV)
**Static (0 K)**	-6.886	-5.903	0.983
**300 K**	-6.874 (+12)	-5.990 (-87)	0.884 (-99)

Supplementary Table S5 | Summarized energy level donor-bridge-acceptor at 0 K. Level alignment of isolated components at the potential energy surface (no temperature). All energies in eV. (intermediate for HSE06 and tight basis sets for PBE). Relative alignment in parenthesis.

Functional	E_F	VBM	CBM	HOMO	LUMO
**HSE06**	-4.737 (0.0)	-6.415 (-1.678)	-4.075 (0.662)	-7.453 (-2.716)	-5.918 (-1.181)
**PBE**	-4.439 (0.0)	-5.875 (-1.436)	-4.146 (0.293)	-6.891 (-2.452)	-5.909 (-1.470)

Section 9. Minimal model to understand T dependence of level occupations

We write the following single-particle donor-bridge-acceptor Hamiltonian in the basis of the isolated systems (commonly called the diabatic basis)

$$H = \epsilon_D|D\rangle\langle D| + \sum_i^2 \epsilon_{B_i}|B_i\rangle\langle B_i| + \epsilon_A \ |A\rangle\langle A| + \sum_i^2 \delta_{DB_i}|D\rangle\langle B_i| + \sum_j^2 \delta_{AB_j}|A\rangle\langle B_j| + \delta_{DA}|D\rangle\langle A|, \quad (6)$$

where ϵ refer to the energy levels of the isolated systems and δ to the diabatic couplings between different levels. Taking the energy ϵ_D to set the chemical potential in this system, we are interested in knowing, upon interaction, what will be the final population of the A state, i.e., what is the CT from D to A *via* B. Upon solution of this model, the eigenvalues ϵ_j and eigenvectors $|j\rangle$ of the interacting system are obtained. We are then interested in calculating the population of the original states after interaction. In a single-particle picture, this can be obtained by

$$p_X = \frac{\langle X|e^{-H/k_\mathrm{B}T}|X\rangle}{\sum_j e^{-\epsilon_j/k_\mathrm{B}T}} = \frac{\sum_j \langle X|j\rangle\langle j|X\rangle}{\sum_j e^{-\epsilon_j/k_\mathrm{B}T}}, \quad (7)$$

where X is the state of interest. In this expression, the chemical potential is implicitly assumed to be at zero of energy. One can thus, e.g., solve the model by placing the Fermi level of graphite (D) at 0 eV and expressing the relative position of the other three levels with respect to it, taking their values at the potential energy surface, as shown in Table S5. We assume that $\delta_{DB_i} = 0.5$, $\delta_{AB_i} = 0.2$ and $\delta_{DA_i} = 0.01\ eV$. These are empirical values but reasonable for the vdW bonded systems regarded here and the distances involved between the components.[18] A first-principles calculation of these coupling parameters is desirable but could not be achieved yet for these systems with the electronic-structure codes we employ. We vary T for the along the temperatures considered in this study.

We then let the position of the VBM of ML-MoS_2 and the acceptor LUMO vary in the range we have calculated in Tables S3 and S4 (we found that varying the CBM made no difference to the results). The variation of the population of the molecular level is shown in Figure S14. This allows us to exemplarily visualize how the population of acceptor state A (LUMO of F6TCNNQ) depends on the energy ϵ_A and the energy of the VBM of ML-MoS_2 (ϵ_{B1}), which both vary as function of temperature as shown in Tables S3 and S4.

We can conclude the following: (i) The direction of the renormalization of the state energies can lead to an appreciable increase of the molecular state population. This is due to the coupling between the different parts of the system and the electronic level renormalization. (ii) The quantitative amount of variation of the population of the molecular levels will depend on the actual values of the coupling.

However, varying the coupling constants within a sensible range (0.1-0.8 eV) does not change the qualitative picture shown in this paper.

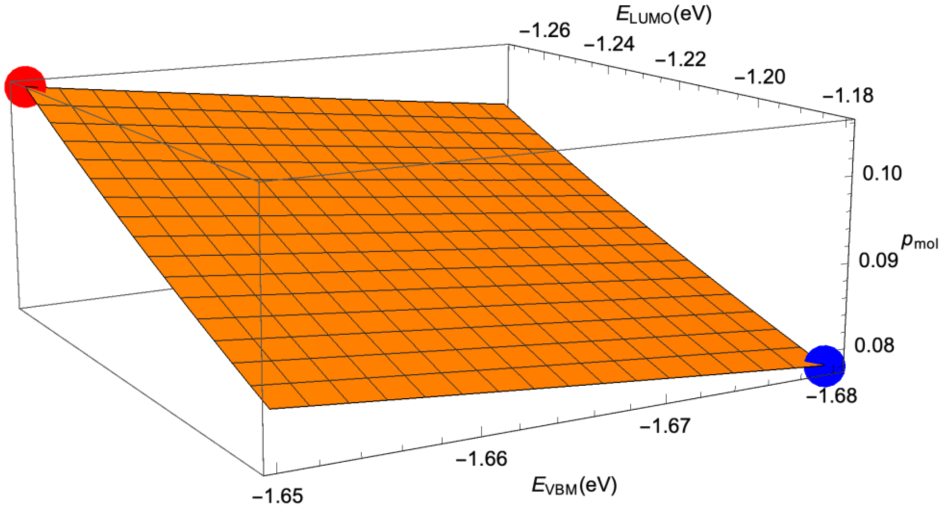

Supplementary Figure S14 | Population of the molecular level upon a linear variation of the relative VBM and LUMO energies. The blue dot corresponds to the values at the potential energy surface and the red dot corresponds to population with the renormalized levels at 300 K.

Reference

[1] S. Park, T. Schultz, X. Xu, B. Wegner, A. Aljarb, A. Han, L. Li, V. C. Tung, P. Amsalem, N. Koch, *Commun. Phys.* **2019**, *2*, 109.

[2] M.-H. Chiu, C. Zhang, H.-W. Shiu, C.-P. Chuu, C.-H. Chen, C.-Y. S. Chang, C.-H. Chen, M.-Y. Chou, C.-K. Shih, L.-J. Li, *Nat. Commun.* **2015**, *6*, 7666.

[3] Z. Y. Zhu, Y. C. Cheng, U. Schwingenschlögl, *Phys. Rev. B* **2011**, *84*, 153402.

[4] D. Xiao, G.-B. Liu, W. Feng, X. Xu, W. Yao, *Phys. Rev. Lett.* **2012**, *108*, 196802.

[5] S. Park, T. Schultz, A. Han, A. Aljarb, X. Xu, P. Beyer, A. Opitz, R. Ovsyannikov, L.-J. Li, M. Meissner, T. Yamaguchi, S. Kera, P. Amsalem, N. Koch, *Commun. Phys.* **2019**, *2*, 68.

[6] C. Christodoulou, A. Giannakopoulos, G. Ligorio, M. Oehzelt, M. Timpel, J. Niederhausen, L. Pasquali, A. Giglia, K. Parvez, K. Müllen, D. Beljonne, N. Koch, M. V. Nardi, *ACS Appl. Mater. Interfaces* **2015**, *7*, 19134.

[7] P. B. Allen, M. Cardona, *Phys. Rev. B* **1981**, *23*, 1495.

[8] Y. Baskin, L. Meyer, *Phys. Rev.* **1955**, *100*, 544.

[9] J. Judek, A. P. Gertych, K. Czerniak, M. Zdrojek, *Phys. Chem. Chem. Phys.* **2018**, *20*, 15486.

[10] F. E. Williams, *Phys. Rev.* **1951**, *82*, 281.

[11] M. Lax, *J. Chem. Phys.* **1952**, *20*, 1752.

[12] M. Zacharias, C. E. Patrick, F. Giustino, *Phys. Rev. Lett.* **2015**, *115*, 177401.

[13] M. Zacharias, F. Giustino, *Phys. Rev. Res.* **2020**, *2*, 013357.

[14] M. Zacharias, F. Giustino, *Phys. Rev. B* **2016**, *94*, 075125.

[15] A. Togo, I. Tanaka, *Scr. Mater.* **2015**, *108*, 1.

[16] S. Joe, F. Y. Kuo, *SIAM J. Sci. Comput.* **2008**, *30*, 2635.

[17] A. Molina-Sánchez, M. Palummo, A. Marini, L. Wirtz, *Phys. Rev. B* **2016**, *93*, 155435.

[18] A. Kubas, F. Gajdos, A. Heck, H. Oberhofer, M. Elstner, J. Blumberger, *Phys. Chem. Chem. Phys.* **2015**, *17*, 14342.
